# Pronounced social inequality in health-related factors and quality of life in women and men from Austria who are overweight or obese

**DOI:** 10.7717/peerj.6773

**Published:** 2019-05-07

**Authors:** Nathalie Tatjana Burkert, Wolfgang Freidl

**Affiliations:** Institute of Social Medicine and Epidemiology, Medical University Graz, Graz, Austria

**Keywords:** Socioeconomic status, Chronic conditions, Overweight, Health-related factors, Body mass index, Health, Quality of life, Obesity

## Abstract

**Background:**

The burden of social inequalities in health as well as the association between obesity with morbidity and mortality is a worldwide problem. Therefore, the aim of our study was to investigate health-related factors, health, and quality of life in Austrian women and men with normal weight, overweight, and obesity with a different socioeconomic status (SES) based on actual data from 2015.

**Methods:**

This representative population-based study was based on self-reported data of 15,338 Austrian adults (8,425 women and 6,933 men) in 2014/2015. Data of the Austrian Health Interview Survey was analyzed stratified by sex and adjusted for age concerning health-related behavior, health, and quality of life.

**Results:**

The results have shown that people with a low SES differ significantly from those of high SES concerning health-related factors (e.g., eating behavior, physical activity), health and impairment due to chronic conditions, as well as quality of life. Obesity in women and men was associated with poorer health-related factors and more chronic conditions as well as unfavorable psychological aspects. In women, the results showed a significant body mass index*SES interaction for impairment due to disorders, the number of chronic conditions and quality of life in the domain of physical health. In men, the interaction was significant regarding alcohol consumption, as well as health impairment. The SES has a strong negative impact on health which implies that people of low SES have more health problems which especially concerns individuals who are obese. Therefore, a continuous target group-oriented, non-discriminatory, interdisciplinary public health program is required, prioritizing women, and men with obesity with a low SES.

## Introduction

The burden of social inequalities in health is a common worldwide problem (Marmot & Wilkinson, 2005). Social and economic resources have an influence on health (World Health Organization (WHO), 2013). Moreover, the socioeconomic status (SES) is recognized as an important determinant associated with several health risk factors (Blakely, Hales & Woodward, 2004). The mortality risk associated with social inequality has risen significantly in the past 30 years in Europe (Menke, Streich & Rössler, 2003). Studies have shown that the SES has a large impact on physical as well as psychological health and subsequently also on mortality and life expectancy in both men and women (Bauer et al., 2009; Freidl et al., 2001; Hoebel et al., 2017; Holstein et al., 2009; Marmot, 2007; Marmot & Wilkinson, 2005; Österreichisches Bundesinstitut für Gesundheitswesen (ÖBIG), 2003). People of low SES have an increased prevalence of morbidity and mortality (Mackenbach, 2006) and a life expectancy lower by 7 years (The Marmot Review, 2010). Studies have also shown that a social gradient in the prevalence of depression exists (Huurre et al., 2007; Van Der Waerden et al., 2014). A low SES that persists over time is related to a higher prevalence of mental health problems (Reiss, 2013). Moreover, obesity is related to a lower mental wellbeing (Stranges et al., 2014). One of the reasons for the difference in ill health and mortality between persons of a different SES, are the distinctions in health related behavior (Österreichisches Bundesinstitut für Gesundheitswesen (ÖBIG), 2003), but also in their stress levels (Yang et al., 2017), work environment (Kristensen, Borg & Hannerz, 2002), and in the use of preventive health care (Cooper et al., 2017; Janßen, Sauter & Kowalski, 2012). Lifestyle factors which include a health risk are tobacco smoking, alcohol consumption, inadequate eating behavior, and physical inactivity (Branca, Nikogosian & Lobstein, 2007). All these risk factors are more present in adults with a low SES (Branca, Nikogosian & Lobstein, 2007; Brennan et al., 2009; Lantz et al., 2010). Smoking, an important contributor to health inequalities, is more common among people of low SES (Mackenbach, 2006). The health risk which is associated with alcohol intake is complex. While excessive drinking is associated with a risk for health (Mackenbach, 2006), moderate drinking seems to be beneficial for health and is associated with a lower mortality rate (Worm, Belz & Stein-Hammer, 2013). Moreover, the risk to suffer from diseases like coronary heart disease seems to be lower in people who consume alcohol moderately (Mathews, Liebenberg & Mathews, 2015). Concerning alcohol consumption an inverse social gradient seems to exist: people with a low SES are more likely to be abstinent, but when they consume alcohol, they more often show problematic drinking patterns (The Marmot Review, 2010). While heavy drinking is also associated with weight-gain, moderate drinking does not seem to be related to obesity (Traversy & Chaput, 2015). Food consumption is also determined by socioeconomic factors. People with a lower SES consume less fruits and vegetables (Paalanen et al., 2011), but more food with higher energy and lower micronutrients (Kuipers, 2010). Additionally, overweight and obesity are associated with insufficient fruit and vegetable intake (Zalewska & Maciorkowska, 2017). Physical activity is inversely associated with SES (Kuipers, 2010). The SES has an influence on medical care of persons with a different social background (Österreichisches Bundesinstitut für Gesundheitswesen (ÖBIG), 2003; Steinkamp, 1999) and also preventive health care is more frequently used by individuals with high SES. Moreover, overweight and obesity are inversely associated with the SES (Marija et al., 2018).

Studies from Austria has also shown social inequality in health and health related behavior (Burkert, Rásky & Freidl, 2012; Burkert et al., 2012, 2013a, 2013b). People with a low SES have a poorer health related behavior—they consume more food with a higher amount of animal fat intake and are less physical active (Burkert, Rásky & Freidl, 2012). Moreover, they report a poorer state of health generally, a higher number of chronic conditions, a lower quality of life, and more impairment due to ill health (Burkert, Rásky & Freidl, 2012). Additionally, health-related factors, like malnutrition, physical inactivity, and subsequently the risk of obesity rises with declining social status (Österreichisches Bundesinstitut für Gesundheitswesen (ÖBIG), 2003; Burkert, Rásky & Freidl, 2012; Großschädl, 2013; Howel et al., 2013; Kiefer et al., 2006; World Health Organization (WHO), 1998). Moreover, with rising income inequality in countries, the prevalence of people who are overweight and obese increases (Wilkinson & Pickett, 2009). Worldwide the prevalence of obesity has more than doubled in the past 35 years (World Health Organization (WHO), 2012). In 2014, more than 1.9 billion people over 18 years of age were overweight, and of these more than 600 million even obese (World Health Organization (WHO), 2016). Overall, more than one million deaths and 12 million life years of illness each year are caused by obesity (Branca, Nikogosian & Lobstein, 2007). The World Health Organization identified overweight and obesity, as well as smoking, alcohol consumption, hypertension, and sexual practices as the main causes of morbidity and mortality worldwide—mainly concerning people of a low SES (World Health Organization (WHO), 2000). Lower ratings of health as well as poor health-related behavior in general seem to be associated with obesity (Burkert, Rásky & Freidl, 2012). With rising body mass index (BMI) the risk of ill health increases: Overweight and obesity are associated with an increased health risk to suffer from diseases like diabetes, hypertension, coronary heart disease, osteoarthritis, or hormone-dependent sorts of cancer (Österreichisches Bundesinstitut für Gesundheitswesen (ÖBIG), 2003; Kiefer et al., 2006; Großschädl et al., 2015). Additionally, obesity is associated with less healthy lifestyle, psychological problems and a lower quality of life (Branca, Nikogosian & Lobstein, 2007; Brennan et al., 2009; Kiefer et al., 2006; Kinge & Morris, 2010). The results from an Austrian study showed that the social status has a greater impact on health related behavior and health in obesity (Burkert et al., 2013a, 2013b). In persons with obesity the social background has the greatest influence on the amount of physical exercise, impairment due to disorders, and the number of chronic conditions (Burkert et al., 2013a). Nevertheless current clinical guidelines and public health statements often link the BMI to diseases, regardless of the individual’s situation (e.g., age, SES, genes) (Kuipers, 2010).

In conclusion, it can be stated that a low SES per se is combined with a higher health risk and a risk factor for morbidity and mortality (Mackenbach, 2006; Kuipers, 2010). Since the prevalence of obesity is increasing worldwide and social inequality in health is a worldwide problem, the aim of this study was to analyze health-related factors, health, medical care, and quality of life in Austrian adults with normal weight, overweight, or obesity with a different socioeconomic background based on actual data from 2015.

## Materials and Methods

### Study population

The analyzed data used for this study was taken from the Austrian Health Interview Survey (ATHIS) 2014/15 (Bundesministerium für Gesundheit (BMG), 2015). The ATHIS is part of the European Health Interview Survey (EHIS; https://ec.europa.eu/eurostat/web/microdata/european-health-interview-survey) which is an important survey of high quality. The study was conducted through computer assisted telephone interviewing (CAPI) as well as a questionnaire which was filled out by the participants.

The study population is representative for the Austrian population. To get a representative sample, the respondents were stratified according to Austria’s different geographic regions, and the sampling was weighted according to the number of the inhabitants in the specific regions. The data were obtained from adults aged 15 years and older (15,771 participants; 55.7% female). All participants were first contacted and informed about the survey by telephone. If they agreed to take part, an appointment for the CAPI was arranged. No minors or children and only those respondents who could provide information personally were included in the study. The response rate of the ATHIS was 40.7%.

Analyses were calculated for people with normal-weight, overweight, and obesity with a low, middle or high SES (*N* = 15,358; 8,425 women and 6,933 men). Descriptive characteristics for the participants included in the analyses are shown in Table 1. Due to missing data concerning the variables “physical activity” and “vaccine protection” the number of people which were analyzed concerning their health-related behavior was 10,583. For the domains “health” and “quality of live” 15,358 adults were included in the analyses. In the footnotes of Tables 2–8 the number of participants which were included in the analyses is shown.

**Table 1:** Descriptive statistics, number of individuals.

	Low SES	Middle SES	High SES	Total
Normal weight				
Women	1,622	1,331	2,101	5,054
15–19	153	37	14	204
20–34	303	374	551	1,228
35–49	400	428	884	1,712
50–64	446	341	550	1,337
65–79	255	136	93	484
80+	65	15	9	89
Men	882	755	1,305	2,942
15–19	148	42	11	201
20–34	187	228	390	805
35–49	202	196	489	887
50–64	172	170	320	662
65–79	145	105	84	334
80+	28	14	11	53
Overweight				
Women	1,001	627	643	2,271
15–19	21	9	2	32
20–34	99	83	108	290
35–49	190	171	248	609
50–64	351	221	226	798
65–79	299	132	52	483
80+	41	11	7	59
Men	914	796	1,211	2,921
15–19	27	12	8	47
20–34	103	114	211	428
35–49	238	237	442	917
50–64	287	276	418	981
65–79	234	143	119	496
80+	25	14	13	52
Obesity				
Women	594	296	210	1,100
15–19	5	0	0	5
20–34	54	33	22	109
35–49	121	76	78	275
50–64	230	133	90	453
65–79	161	47	18	226
80+	23	7	2	32
Men	424	300	346	1,070
15–19	7	3	1	11
20–34	45	46	48	139
35–49	103	82	101	286
50–64	157	102	159	418
65–79	106	64	32	202
80+	6	3	5	14
Total	5,437	4,105	5,816	15,358

**Table 2:** Health behaviour in normal weight, overweight, and obese women.

	Low SES		Middle SES		High SES		Test statistics		
	M	SD	M	SD	M	SD	BMI	SES	BMI*SES
Health behavior							**0.000**	**0.000**	0.240
Normal weight									
Healthy eating behavior^1^	1.88	0.36	1.86	0.34	1.81	0.32	**0.000**	**0.000**	0.450
Smoking behavior^2^	2.93	6.06	2.58	5.75	1.81	4.89	0.136	**0.034**	0.120
Alcohol consumption^1^	5.98	1.83	5.61	1.87	5.19	1.71	**0.000**	**0.000**	0.240
Physical activity at work^3^	2.07	0.94	1.83	0.87	1.45	0.68	**0.047**	**0.000**	0.326
Physical activity during leisure time^4^	3.38	1.82	3.36	1.79	3.25	1.66	**0.000**	0.194	0.561
Number of vaccine protections	2.35	1.47	2.65	1.45	2.99	1.38	**0.008**	**0.000**	0.316
Overweight									
Healthy eating behavior	1.88	0.32	1.85	0.31	1.82	0.30			
Smoking behavior	2.27	5.38	2.05	5.62	2.09	5.23			
Alcohol consumption	5.93	1.89	5.68	1.76	5.45	1.69			
Physical activity at work	2.27	0.99	2.02	0.94	1.55	0.79			
Physical activity during leisure time	3.45	1.86	3.19	1.79	3.15	1.72			
Number of vaccine protections	2.43	1.48	2.57	1.50	3.02	1.35			
Obesity									
Healthy eating behavior	1.89	0.33	1.92	0.32	1.88	0.34			
Smoking behavior	2.65	6.34	2.42	6.73	2.81	5.93			
Alcohol consumption	6.36	1.79	6.22	1.96	5.59	1.70			
Physical activity at work	2.36	1.08	2.01	0.97	1.52	0.76			
Physical activity during leisure time	3.23	1.88	2.99	1.62	3.05	1.54			
Number of vaccine protections	2.34	1.53	2.65	1.49	2.86	1.45			

**Notes:**

Data source: Austrian Health Interview Survey (ATHIS) 2014/2015; *n* = 5,928 women. M, mean; SD, standard deviation. Analyses were calculated stratified by sex and controlled for age. The significant levels are bolded.

1 A lower value means a higher intake.

2 A lower value means less consume.

3 A lower value means less activity.

4 Number of days and a lower number means less support.

**Table 3:** Health behaviour in normal weight, overweight, and obese men.

	Low SES		Middle SES		High SES		Test statistics		
	M	SD	M	SD	M	SD	BMI	SES	BMI*SES
Health behavior							**0.000**	**0.000**	0.065
Normal weight									
Healthy eating behavior^1^	2.08	0.35	2.04	0.37	1.99	0.33	**0.003**	**0.013**	0.121
Smoking behaviour^2^	4.47	8.60	4.11	7.91	2.72	6.26	0.354	**0.000**	0.399
Alcohol consumption^1^	4.81	2.10	4.68	1.87	4.27	1.75	**0.007**	**0.000**	**0.043**
Physical activity at work^3^	2.03	0.92	1.96	0.91	1.41	0.74	0.102	**0.000**	0.060
Physical activity during leisure time^4^	3.44	1.74	3.52	1.83	3.36	1.71	**0.000**	**0.001**	0.249
Number of vaccine protections	2.59	1.36	2.67	1.40	3.02	1.35	0.948	**0.000**	0.326
Overweight									
Healthy eating behavior	2.03	0.36	2.04	0.33	2.03	0.34			
Smoking behavior	3.75	8.46	4.04	7.82	2.88	6.90			
Alcohol consumption	4.85	2.08	4.35	2.01	4.19	1.76			
Physical activity at work	2.25	0.93	2.06	0.90	1.43	0.76			
Physical activity during leisure time	3.37	1.86	3.23	1.64	3.12	1.68			
Number of vaccine protections	2.39	1.42	2.62	1.39	3.00	1.38			
Obesity									
Healthy eating behavior	2.04	0.33	2.05	0.34	2.04	0.33			
Smoking behavior	3.25	8.88	4.78	9.38	3.22	7.44			
Alcohol consumption	4.81	2.20	4.66	2.14	4.48	1.86			
Physical activity at work	2.38	1.08	2.10	0.97	1.44	0.78			
Physical activity during leisure time	3.38	1.76	3.17	1.83	2.83	1.49			
Number of vaccine protections	2.26	1.51	2.70	1.34	3.06	1.33			

**Notes:**

Data source: Austrian Health Interview Survey (ATHIS) 2014/2015; *n* = 4,655 men. M, mean; SD, standard deviation. Analyses were calculated stratified by sex and controlled for age. The significant levels are bolded.

1 A lower value means a higher intake.

2 A lower value means less consume.

3 A lower value means less activity.

4 Number of days and a lower number means less support.

**Table 4:** Health in normal weight, overweight, and obese women.

	Low SES		Middle SES		High SES		Test statistics		
	M	SD	M	SD	M	SD	BMI	SES	BMI*SES
Health							**0.000**	**0.000**	**0.002**
Normal weight									
Self-reported health generally^1^	1.97	0.88	1.70	0.76	1.53	0.66	**0.000**	**0.000**	0.991
Impairment^2^	2.59	0.62	2.70	0.54	2.74	0.50	**0.000**	**0.000**	**0.008**
Chronic conditions^1^	1.46	1.69	1.13	1.32	0.96	1.19	**0.000**	**0.000**	**0.003**
Overweight									
Self-reported health generally	2.24	0.90	1.95	0.74	1.72	0.69			
Impairment	2.43	0.69	2.65	0.56	2.69	0.55			
Chronic conditions	2.06	1.95	1.54	1.51	1.27	1.43			
Obesity									
Self-reported health generally	2.52	0.92	2.24	0.87	2.03	0.73			
Impairment	2.24	0.74	2.41	0.67	2.55	0.62			
Chronic conditions	2.72	2.24	2.25	1.94	1.67	1.62			

**Notes:**

Data source: Austrian Health Interview Survey (ATHIS) 2014/2015; *n* = 8,425 women. M,mean; SD, standard deviation. Analyses were calculated stratified by sex and controlled for age. The significant levels are bolded.

1 A lower value means better health.

2 A lower value means more impairment.

**Table 5:** Impairment due to disorders in normal weight, overweight, and obese women and men with a different SES.

Impairment due to disorders						
	Women			Men		
	Highly impaired (%)	Impaired (%)	Not impaired (%)	Highly impaired (%)	Impaired (%)	Not impaired (%)
Normal weight						
Low SES	6.9	27.4	65.7	6.9	23.6	69.5
Middle SES	4.2	21.9	73.9	4.1	20.9	75.0
High SES	2.9	20.2	76.9	1.5	16.2	82.2
Overweight						
Low SES	11.3	34.0	54.7	9.0	29.0	62.0
Middle SES	4.1	26.6	69.2	6.0	26.6	67.3
High SES	4.0	23.3	72.6	3.0	19.1	78.0
Obesity						
Low SES	18.0	40.2	41.8	18.4	33.5	48.1
Middle SES	10.5	38.5	51.0	8.3	30.7	61.0
High SES	6.7	31.9	61.4	4.6	27.5	67.9

**Table 6:** Health in normal weight, overweight, and obese men.

	Low SES		Middle SES		High SES		Test statistics		
	M	SD	M	SD	M	SD	BMI	SES	BMI*SES
Health							**0.000**	**0.000**	0.065
Normal weight									
Self-reported health generally^1^	1.90	0.88	1.75	0.76	1.53	0.65	**0.000**	**0.000**	0.236
Impairment^2^	2.63	0.61	2.71	0.54	2.81	0.43	**0.000**	**0.000**	**0.012**
Chronic conditions^1^	1.15	1.48	0.93	1.17	0.75	1.01	**0.000**	**0.000**	0.197
Overweight									
Self-reported health generally	2.09	0.84	1.93	0.79	1.70	0.69			
Impairment	2.53	0.66	2.61	0.60	2.75	0.50			
Chronic conditions	1.57	1.64	1.29	1.42	0.94	1.18			
Obesity									
Self-reported health generally	2.47	0.95	2.18	0.88	2.01	0.74			
Impairment	2.30	0.76	2.53	0.65	2.63	0.57			
Chronic conditions	2.18	2.07	1.67	1.63	1.57	1.47			

**Notes:**

Data source: Austrian Health Interview Survey (ATHIS) 2014/2015; *n* = 6,933 men. M, mean; SD, standard deviation. Analyses were calculated stratified by sex and controlled for age. The significant levels are bolded.

1 A lower value means better health.

2 A lower value means more impairment.

**Table 7:** Quality of life in normal weight, overweight and obese women.

	Low SES		Middle SES		High SES		Test statistics		
	M	SD	M	SD	M	SD	BMI	SES	BMI*SES
Quality of life							**0.000**	**0.000**	0.375
Normal weight									
WHOQOL physical health^1^	16.37	2.87	17.15	2.35	17.52	2.07	**0.000**	**0.000**	**0.029**
WHOQOL psychological health^1^	15.74	2.35	16.22	2.20	16.65	2.04	**0.000**	**0.000**	0.494
WHOQOL social relationships^1^	16.15	2.76	16.36	2.64	16.63	2.55	**0.002**	**0.001**	0.869
WHOQOL environment^1^	16.07	2.24	16.63	2.01	17.14	1.81	**0.000**	**0.000**	0.921
Overweight									
WHOQOL physical health	15.75	2.86	16.75	2.35	17.14	2.32			
WHOQOL psychological health	15.50	2.31	16.04	2.13	16.36	2.14			
WHOQOL social relationships	15.92	2.69	16.15	2.49	16.42	2.42			
WHOQOL environment	15.87	2.17	16.50	1.98	16.95	1.96			
Obesity									
WHOQOL physical health	14.83	3.27	15.84	2.77	16.73	2.51			
WHOQOL psychological health	14.89	2.62	15.52	2.30	16.08	2.26			
WHOQOL social relationships	15.56	2.75	15.93	2.75	16.03	2.80			
WHOQOL environment	15.53	2.31	16.15	2.06	16.74	2.09			

**Notes:**

Data source: Austrian Health Interview Survey (ATHIS) 2014/2015; *n* = 8,425 women. M, mean; SD, standard deviation. Analyses were calculated stratified by sex and controlled for age. The significant levels are bolded.

1 A lower value means a lower quality of life.

**Table 8:** Quality of life in normal weight, overweight, and obese men.

	Low SES		Middle SES		High SES		Test statistics		
	M	SD	M	SD	M	SD	BMI	SES	BMI*SES
Quality of life							**0.000**	**0.000**	0.126
Normal weight									
WHOQOL physical health^1^	16.72	2.76	17.13	2.35	17.74	1.87	**0.000**	**0.000**	0.107
WHOQOL psychological health^1^	16.36	2.25	16.61	2.13	17.07	1.85	**0.000**	**0.000**	0.125
WHOQOL social relationships^1^	16.02	2.63	16.14	2.52	16.24	2.42	**0.000**	0.201	0.958
WHOQOL environment^1^	16.37	2.10	16.62	1.97	17.31	1.70	**0.000**	**0.000**	0.451
Overweight									
WHOQOL physical health	16.19	2.72	16.61	2.37	17.35	2.15			
WHOQOL psychological health	16.19	2.23	16.51	2.15	16.80	1.99			
WHOQOL social relationships	15.89	2.58	16.04	2.60	16.14	2.56			
WHOQOL environment	16.12	2.10	16.50	1.96	17.11	1.85			
Obesity									
WHOQOL physical health	15.03	3.12	15.98	2.76	16.51	2.42			
WHOQOL psychological health	15.55	2.42	16.13	2.31	16.14	2.20			
WHOQOL social relationships	15.35	2.51	15.52	2.81	15.50	2.70			
WHOQOL environment	15.63	2.05	16.21	2.08	16.70	1.99			

**Notes:**

Data source: Austrian Health Interview Survey (ATHIS) 2014/2015; *n* = 6,933 men. M, mean; SD, standard deviation. Analyses were calculated stratified by sex and controlled for age.

1 A lower value means a lower quality of life.

### Measurements

Telephone interviews were conducted, questioning the participants about socio-demographic characteristics, their health, health-related behavior, and psychological aspects.

The independent variables in this study were the weight class (normal-weight, overweight, or obesity) and the SES (ranging from 0 to 15). All individuals self-reported their actual weight and height. The BMI was calculated by dividing the weight of the person in kilogram (kg) by their squared height in meters (m; kg/m^2^). The classification for normal weight, overweight, and obesity were made according to the World Health Organization (WHO) (2000) (underweight (BMI < 18.5 kg/m^2^), normal-weight (BMI ≥ 18.5 kg/m^2^ and <25 kg/m^2^), overweight (BMI ≥ 25 kg/m^2^ and <30 kg/m^2^), or obese (30 kg/m^2^ or more)).

The SES was calculated using the following three variables: net equivalent income, level of education, and occupation. Net equivalent income was calculated based on an equivalence scale provided by the Organization for Economic Co-operation and Development (OECD) (OECD Social Policy Division, 2009), and was determined by dividing the net income of the respondent by the sum of the equivalent weights of all members of her/his household as follows: a weight of 1.0 was assigned to the head of the household, weights of 0.5 to additional members who were 14 years or older, and weights of 0.3 to children who were younger than 14 years of age. The net equivalent income of all respondents was then categorized into quintiles, leading to scores ranging from one to five. Level of education was measured by an ordinal variable distinguishing between (1) basic education (up to 15 years of age), (2) apprenticeship/vocational school, (3) secondary education without diploma, (4) secondary education with diploma, and (5) university. The occupation of the participants was also differentiated into five different levels: (1) unskilled worker, (2) apprentice/skilled worker, (3) self-employed/middle job, (4) qualified job/academic, (5) executive position. To verify the combination of factors that served to calculate the SES, correlations with the different factors were calculated which showed a significant association between all variables. The SES was then divided into three classes: a low (SES ≤ 6), middle (SES = 7,8) or high (SES ≥ 9) SES, based on percentile 33.33 and 66.67.

Weight has a different influence on health in men and women (Hemmelmann et al., 2010; Hüsler et al., 2010), we therefore analyzed the data stratified by sex. Additionally, we analyzed the data by controlling for the age of the participants since also age is related to health (Dorner & Rieder, 2010; Volkert, 2006).

The data was analyzed for three different dimensions: health-related factors, ill health, and quality of life. The dependent variables concerning health-related behavior were the eating behavior concerning fruit, vegetable, fish, and meat consumption. Participants were asked how often they eat the above mentioned kinds of food (every day, three to four times per week, once or twice per week, once or twice per month, less than once a month, or never). The mean value of a high fruit, vegetable, and fish consumption and a low meat intake was calculated to analyse healthy eating (1–6; interval scale; higher value means a healthier eating style; for comparison: https://ec.europa.eu/eurostat/web/microdata/european-health-interview-survey). Further health-related variables which were analyzed were smoking behavior (cigarettes per day; interval scale), alcohol consumption during the last year (consuming alcohol every day, five to six times per week, three to four times per week, once or twice per week, two or three times per month, once in a month, less than once in a month, never during the past year, or never ever; interval scale), and the number on days during the last week on which physical exercise of more than 10 min per day was practiced (for comparison: https://ec.europa.eu/eurostat/web/microdata/european-health-interview-survey). Additionally, the number of active vaccinations (influenza, tetanus, diphtheria, polio, and early summer meningo-encephalitis) was analyzed by calculating a sum index concerning the five different vaccinations (0–5, sum index; interval scale), coded as present (1) or absent (0).

The dependent variables focusing on ill-health included impairment to health, ranging from one (not impaired) to three (highly impaired; ordinal data) and self-perceived health, ranging from one (very good) to five (very bad), and. The presence of 17 chronic conditions (asthma, bronchitis, heart infarction, coronary heart disease, hypertension, stroke, arthritis, lower and higher back pain, diabetes, allergies, hepatic cirrhosis, urinary incontinency, kidney problems, depression, migraine, and gastrointestinal ulcer) was assessed. Each condition was coded as present (1) or absent (0). The total frequency score was calculated by summing up the present chronic conditions (0–17, sum index). All variables (self perceived health and the number of chronic conditions) are based on an interval scale and were also used as part of the EHIS (for comparison: https://ec.europa.eu/eurostat/web/microdata/european-health-interview-survey).

The dependent variables concerning quality of life were measured using the short version of the World Health Organization Quality of Life Questionnaire (WHOQOL-BREF) (World Health Organization (WHO), 1996). The four domain scores (physical health, psychological health, social relationships, and environment) were calculated (interval scale; Kalfoss, Low & Molzahn, 2008; Skevington, Lotfy & O’Connell, 2004; Skevington & McCrate, 2012). Domains ranged between a score of 4 and 20.

### Statistical analyses

We analyzed a 3 × 3 design based on people with a different BMI (normal-weight (BMI between 18.5 and 25 kg/m^2^), overweight (BMI between 25 and 30 kg/m^2^), and obesity (from 30 kg/m^2^ up)) and various SES (low, middle or high one). The obtained data was analyzed stratified by sex and controlled for the age of the participants. The total number of participants analyzed was 15,358 (8,425 women and 6,933 men, see Table 1).

In order to analyze the differences and variation between individuals with a different weight status and SES, in terms of health behavior, ill health, and quality of life, multivariate analyses of variance were conducted for each domain corrected for the age of the individuals separately for women and men. Tukey HSD was used as post-test. *P*-values < *0.05* were considered statistically significant. Since impairment due to diseases was only measured by differentiating between highly impaired, impaired, and not impaired, the results were also calculated by means of Chi-square tests. Nevertheless, since we analyzed a large number of participants results of both measures are reported. All analyses were calculated using SPSS software (version 25.0).

## Results

### General characteristics

In 2014/2015, nearly half of the women with a low SES were normal-weight (50.4%), one third overweight (31.1%), and about a fifth obese (18.5%). Almost two out of three women with a middle SES were normal-weight (59.1%), more than one quarter of them overweight (27.8%) and 13.1% obese. In women with a high SES almost three out of four individuals were normal-weight (71.1%), one fifth was overweight (21.8%) and 7.1% obese.

Overall, nearly two out of five men with a low SES were normal-weight (39.7%), another two out of five were overweight (41.2%) and one fifth obese (19.1%). Also two out of five men with a middle SES were normal-weight (40.8%), 43.0% overweight, and 16.2% obese. Nearly half of all males with a high SES were normal-weight (45.6%), two out of five overweight (42.3%), and only one 10th obese (12.1%).

More than half of the Austrian population reported to consume fruits on a daily basis (60%), while about half of the population consumed vegetables daily, and nearly 60% ate fish once or twice a week. However, almost one quarter also consumed meat on a daily basis. Nevertheless, the analysis concerning the eating behavior in people with obesity showed that a lower percentage consumed fruits (55%) and vegetables (45%) every day compared to the overall Austrian population, while more persons who were obese ate meat every day (30%) than people with a normal-weight (23%).

### Health behavior of Austria women

Results of the multivariate analyses of variance have shown a significant main effect for both the BMI (*p* = 0.000) and SES (*p* = 0.000). Women who were obese consumed less healthy food compared to females with normal-weight (*p* = 0.000). The alcohol consumption was significantly higher in women with normal- and overweight compared to people who were obese (*p* = 0.000). Women with obesity did less physical exercise during their leisure time compared to women with a weight in the normal range (*p* = 0.000). Women who were normal-weight were vaccinated against more diseases than females who were obese (*p* = 0.008).

Women with a low and middle SES showed a less healthy eating behavior than those with a high SES (*p* = 0.000). Females with a low SES smoked significantly more (*p* = 0.034), and drank less alcohol (*p* = 0.000), than those with a middle or high SES. Women with a high SES were vaccinated against more diseases than women with as middle or low SES (*p* = 0.000).

No significant interaction between the BMI and SES was found for women in their health-related behavior. All results are shown in Table 2.

### Health behavior of Austria men

The multivariate analyses of variance have shown a significant main effect for both the BMI (*p* = 0.000) and SES (*p* = 0.000). Men who were overweight and obese showed a significantly unhealthier eating behavior compared to men with normal-weight (*p* = 0.003). The alcohol consumption was significantly higher in men with overweight compared to men who were obese (*p* = 0.007). Overweight and obesity were associated with less physical activity at leisure time compared to normal-weight (*p* = 0.000).

Men with a low SES consumed significantly less healthy food (*p* = 0.013) and did less physical activity during leisure time (*p* = 0.001) than those with a high SES. Austrian men with a low or middle SES smoked more cigarettes per day (*p* = 0.000), and drank less alcohol (*p* = 0.000) compared to those with a high SES.

**Figure 1:**
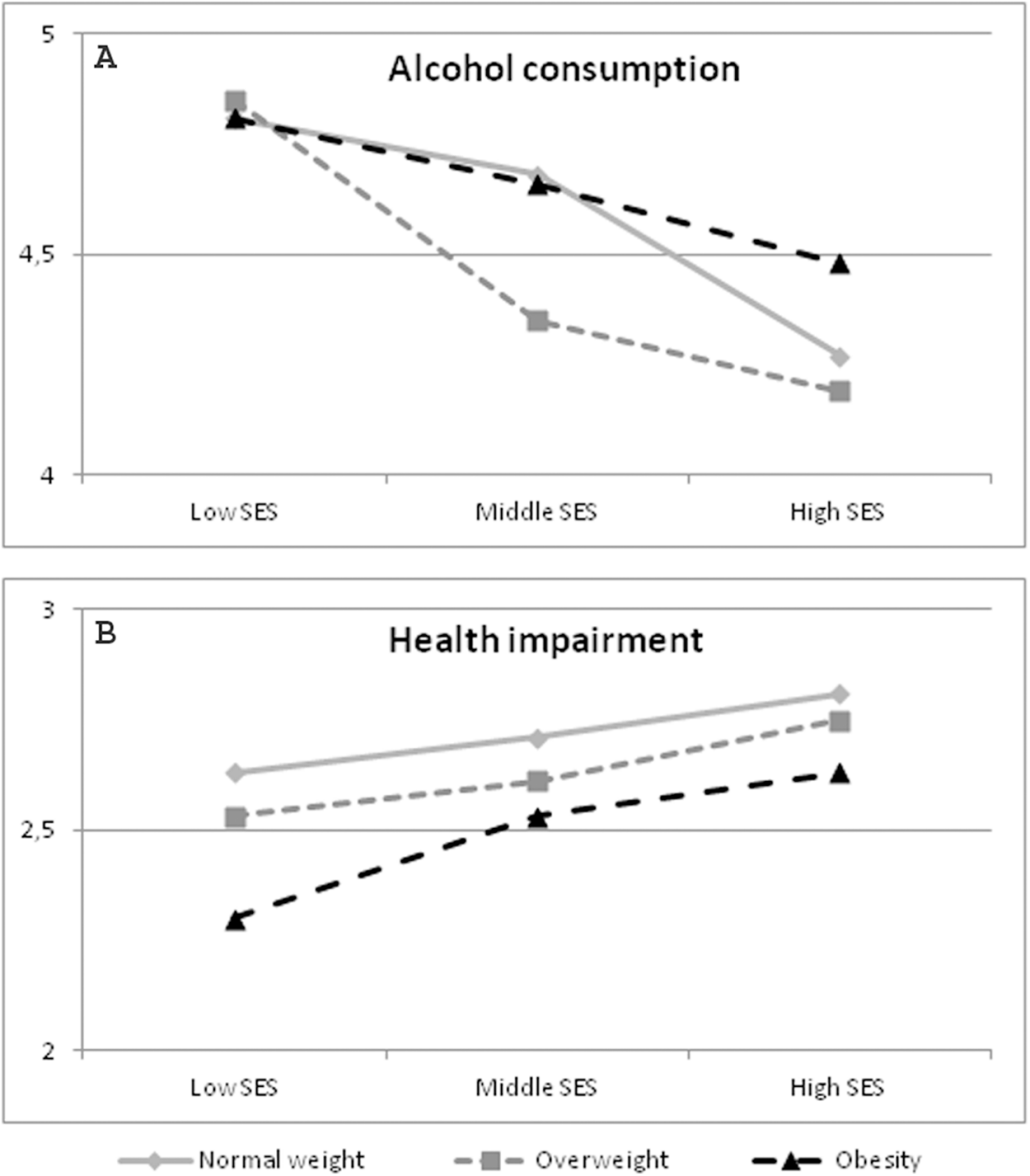
Significant Interactions BMI*SES in men. Data source: Austrian Health Interview Survey (ATHIS) 2014/2015; *n* = 6,933 men. Alcohol consumption: a lower value means more consume; Health impairment: a lower value means more impairment. (A) Significant interaction BMI*SES concerning alcohol consumption (health behavior of Austrian men). (B) Significant interaction BMI*SES regarding health impairment (health of Austrian men).

The results of multivariate test did not show a significant interaction between the BMI and SES for men in Austria (*p* = 0.065). However, according to the univariate analyses, an interaction between the BMI and SES was significant for alcohol intake. The results reveal that in men with overweight the SES has less impact on alcohol consumption, while in obesity especially for men with a high SES the social status seems to have more impact on alcohol intake than in those with a low or middle SES (*p* = 0.043). These results are visualized in Fig. 1A. All results are shown in Table 3.

### Health of Austrian women

For the domain of health, the multivariate analysis of variance in women showed a significant main effect for both the BMI (*p* = 0.000) and SES (*p* = 0.000), as well as a significant interaction (SES*BMI; *p* = 0.002). Women who were overweight and obese reported to be generally in a poorer state of health (*p* = 0.000), to be suffering from more impairment due to disorders (*p* = 0.000), and had more chronic diseases (*p* = 0.000) than persons with normal weight. No impairment in health was reported by nearly three out of four women with normal-weight (72.5%), by about three out of five females who were overweight (63.8%) and only by about half of women with obesity (48.0%; χ^2^ = 294.91, *p* = 0.000).

Women with a low or middle SES reported worse health (*p* = 0.000), more impairment (*p* = 0.000), and suffered from significantly more chronic diseases (*p* = 0.000). About three quarter of all women with a high SES reported to be not impaired in their health (74.9%), also seven out of 10 females with a middle SES stated no impairment (69.6%) and more than half of all women with a low SES reported to be not impaired (57.9%; χ^2^ = 253.79, *p* = 0.000).

**Figure 2:**
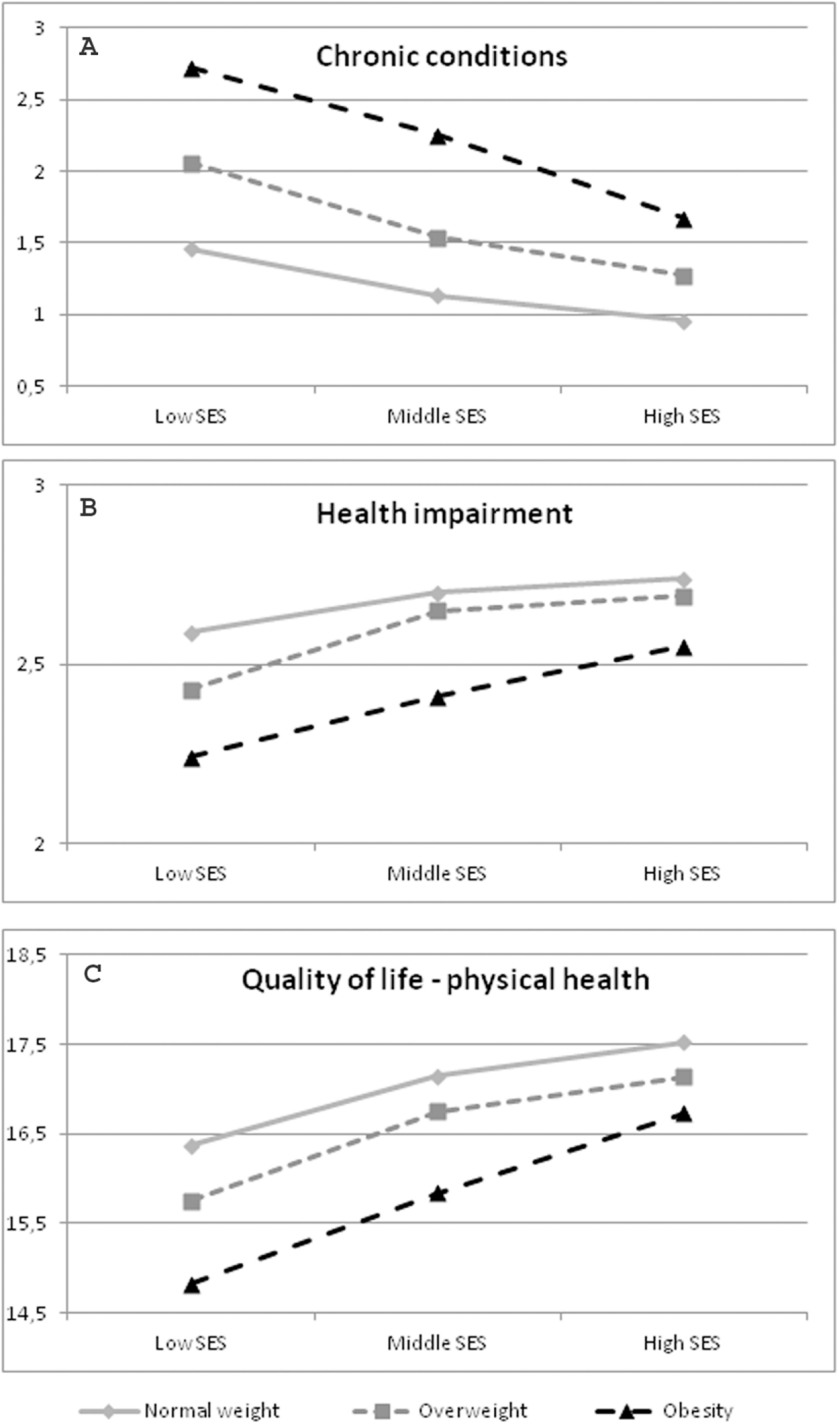
Significant Interactions BMI*SES in women. Data source: Austrian Health Interview Survey (ATHIS) 2014/2015; *n* = 8,425 women. Health impairment: a lower value means more impairment; Chronic conditions: a lower value means better health; Quality of life; a lower value means a lower quality of life. (A) Significant interaction BMI*SES concerning the number of chronic conditions (health of Austrian women). (B) Significant interaction BMI*SES regarding health impairment (health of Austrian women). (C) Significant interaction BMI*SES concerning quality of life in the dimension of physical health (quality of life of Austrian women).

The results showed a significant BMI*SES interaction for impairment due to disorders (*p* = 0.008), as well as for the number of chronic conditions (*p* = 0.003). For both health-related factors the SES had more impact in obesity than in normal-weight. This result is visualized in Figs. 2A and 2B. All results are shown in Table 4. The percentage of women suffering from impairment with a different BMI and SES are shown in Table 5.

### Health of Austrian men

For the domain of health, the multivariate analysis of variance in men also showed a significant main effect for the BMI (*p* = 0.000) and SES (*p* = 0.000). Men with overweight and obesity were in a poorer state of health (*p* = 0.000), suffered from more impairment due to disorders (*p* = 0.000), and reported to suffer from more chronic diseases (*p* = 0.000) than men with normal weight. About three quarter of all men who were normal-weight reported to be not impaired in their health (76.5%), seven out of 10 men with overweight stated no impairment (70.1%) and two out of three males who were obese reported to be not impaired (58.1%; χ^2^ = 253.79, *p* = 0.000).

Men with a high SES reported to be generally in a better state of health (*p* = 0.000), to be suffering from less impairment due to disorders (*p* = 0.000), and to have less chronic diseases (*p* = 0.000) than those with a middle or low SES. No impairment in health was reported by nearly four out of five men with a high SES (78.7%), by about seven out of 10 males with a middle SES (69.4%) and by three out of five men with a low SES (62.3%; χ^2^ = 214.36, *p* = 0.000).

The results of multivariate test showed a significant BMI*SES interaction for impairment due to disorders (*p* = 0.012). In men with obesity the SES had more impact on impairment due to disorders than in men with a normal-weight or who were overweight (Table 5; Fig. 1B). All results are shown in Table 6.

### Quality of life of Austrian women

Regarding the quality of life, the main effect for BMI (*p* = 0.000) and SES (*p* = 0.000) showed a statistically significant effect. Women with obesity had the worst quality of life in the domains physical health (*p* = 0.000), psychological health (*p* = 0.000), social relationships (*p* = 0.002), and also environment (*p* = 0.000).

Moreover, women with a low SES had the lowest quality of life in all four domains of the WHOQOL-bref (physical health: *p* = 0.000, psychological health: *p* = 0.000, social relationships: *p* = 0.001, environment: *p* = 0.000).

Although the MANOVA did not show a significant interaction (*p* = 0.375), the results of the analyses of variance in the different domains showed that in the subtest “physical health” the SES had more impact in adults with an enhanced BMI (*p* = 0.029; Fig. 2C). All results are visualized in Table 7.

### Quality of life of Austrian men

Regarding the quality of life, the main effect for BMI (*p* = 0.000) and SES (*p* = 0.000) also showed a statistically significant effect in Austrian men. Men with obesity stated a worse quality of life in all four domains (physical health: *p* = 0.000, psychological health: *p* = 0.000, social relationships: *p* = 0.000, environment: *p* = 0.000).

Moreover, men with a low SES reported a lower quality of life in the domains physical health (*p* = 0.000), psychological health (*p* = 0.000), and environment (*p* = 0.000).

The SES*BMI interaction was not significant for quality of life for Austrian men (*p* = 0.126). All results are reported in Table 8.

## Discussion

Overall, the findings of this study have shown that a low SES as well as a BMI in the overweight and obese range is associated with worse health-related behavior, poorer health, and lower quality of life. Moreover, our results reveal that in obesity a low SES seems to have more impact on ill health and quality of life regarding physical health, especially in Austrian women.

The health risk of smoking is evident (Branca, Nikogosian & Lobstein, 2007). In 2014/2015 nearly one quarter of the Austrian population reported to smoke. Besides many public health programs which have been implemented to reduce smoking in Austria, the prevalence of smoking was nearly the same as in 2006/2007. Our results have shown that women and men with a low SES smoke more cigarettes per day than those with a higher one, which is in line with previous studies (Branca, Nikogosian & Lobstein, 2007; Brennan et al., 2009; Lantz et al., 2010; Mackenbach, 2006). Moreover, previous studies have shown that the eating behavior has an influence on health (McEvoy, Temple & Woodside, 2012; Singh, Sabaté & Fraser, 2003). Previous findings have also revealed that a low SES as well as obesity is associated with a worse eating behavior (e.g., less fruits and vegetables consume, a higher caloric intake; Burkert et al., 2013a; Kuipers, 2010; Paalanen et al., 2011; Zalewska & Maciorkowska, 2017). The findings of this study have also revealed that a low SES, is associated with worse eating behavior. Unfortunately, the measurement of eating behavior in 2006/2007 in Austria did not cover the same questions as in 2014/2015. Therefore, ascertained comparisons are not possible.

In Industrial countries physical activity at work is minimized, and most jobs comprise sedentary occupations these days (Deutsche Gesellschaft für Ernährung (DGE), 1998). Results of previous studies have shown that men with a lower SES are more physically active at work, but less so during leisure time (Brennan et al., 2010), and that obesity is related to lower levels of non-leisure physical activities (Ahn et al., 2012). Also, the results of this study have shown that obesity as well as a low SES is associated with less physical activity during leisure time. In contrast to previous findings from Austria we could not show that the SES has a stronger impact on physical activity amongst women and men who are obese compared to people with a normal-weight (Burkert et al., 2013a). One explanation for this fact could be the difference in measurement of physical activity. However, further studies especially focusing on movement behavior are necessary to analyse this in more detail. Additionally, our results evidence that women and men with a low SES are vaccinated against fewer diseases.

This study has shown that people with a low SES have a higher mean BMI compared to women and men with a middle and high SES. Moreover, there seems to be a linear association between the BMI and social status in Austria. While only 12% of Austrian men with a high SES are obese, the prevalence in men with a low SES is as high as 19%. In women, 19% with a low SES have a BMI greater than 30 kg/m^2^, while only 7% of women with a high SES are obese. The same association between the prevalence of overweight with the social background of people can be found based on our data. This emphasizes the need for public health problems focusing on women and men with a low SES.

In our study, people of high SES drink alcohol more often than those of low SES. The association between alcohol consumption and health is a complex factor when assessing health risk. Moderate alcohol intake is related to a lower mortality rate (Worm, Belz & Stein-Hammer, 2013) and less weight-gain compared to heavy drinking (Traversy & Chaput, 2015), and previous studies have shown that people of a low SES are more likely to be abstinent, but when they consume alcohol, they more often show problematic drinking patterns (The Marmot Review, 2010). The results of this study have shown that women and men with a low SES overall consume alcohol less regularly. Moreover, our findings revealed that the SES might have a higher impact on alcohol intake in men who were overweight. These findings might indicate that people with a high SES are more likely to drink alcohol moderately, which goes along with reduced mortality rates (Worm, Belz & Stein-Hammer, 2013) and that the health risk linked to alcohol intake might be higher for the low-SES group. However, this fact needs to be analyzed in more in-depth studies.

Previous studies have shown that a low SES is associated with worse health, an enhanced prevalence of diseases (Bauer et al., 2009; Burkert, Rásky & Freidl, 2012; Burkert et al., 2012, 2013a, 2013b; Freidl et al., 2001; Holstein et al., 2009; Mackenbach, 2006; Marmot, 2007; Marmot & Wilkinson, 2005; Menke, Streich & Rössler, 2003; Österreichisches Bundesinstitut für Gesundheitswesen (ÖBIG), 2003), and a life expectancy lower by 7 years (The Marmot Review, 2010). Additionally, overweight and obesity are also related to an increased health risk to suffer from diseases (Burkert et al., 2012, 2013a, 2013b; Großschädl et al., 2015; Kiefer et al., 2006; Österreichisches Bundesinstitut für Gesundheitswesen (ÖBIG), 2003) and more mental health problems (Stranges et al., 2014). The results of this study have shown that a low SES as well as a body weight in the obese category is associated with worse self-perceived health, a higher number of chronic conditions as well as more impairment due to ill health. Studies have shown that self-perceived health is a reliable, valid and efficient measure of physical and mental health (Bacak & Ólafsdóttir, 2017) and a predictor for mortality (Bamia et al., 2017). Therefore, the morbidity and mortality risks might be higher for individuals who are obese as well as women and men with a low SES. Although almost 80% of the Austrian population in 2014/2015 report to be in very good or good health, nearly one quarter reports to suffer from back pain impartial of their weight. Considering only women and men with obesity, even 35% report low or high back pain. Moreover, analyses of the ATHIS in 2014/2015 showed that one fifth of the whole population suffers from hypertension. In obesity, the prevalence of a too high blood pressure is even higher (45%). Since hypertension is associated with a higher health risk, for example, for cardiac problems (Hermann-Lingen, Albus & Titscher, 2008), interventions to reduce the blood pressure in Austrians with obesity are needed.

Most importantly, our findings show that the impact of the social background on health is higher for people with obesity than for adults who are normal-weight and overweight. In women with obesity the social background has the greatest influence on the number of chronic conditions, on impairment due to disorders, and the quality of life regarding physical health. In Austrian men, the impact of the SES on health in obesity was only significant regarding impairment due to disorders; the significant interaction regarding health-related behavior mainly concerned people who were overweight. Since our findings show that the number of chronic diseases is increased in persons with obesity of low SES, they emphasize the influence of socioeconomic factors on the health of individuals with obesity, especially women.

Previous findings revealed that the impact of obesity on self-perceived health is greater among women of low educational level (García-Mendizábal et al., 2009) or low SES (Burkert et al., 2013a, 2013b). In an Austrian study analyzing data from the year 2006/2007, the impact of the SES on health was higher in obesity concerning the number of chronic conditions, impairments in health as well as overall quality of life (Burkert et al., 2013a). Our results are in line with these findings, but mainly concern Austrian women. A study from New Zealand did not show any associations between the SES and quality of life in men with overweight (Derraik et al., 2018). It therefore seems necessary to implement public health programs for people with a low SES prioritizing women who are obese to reduce social inequality in health.

Our findings also showed that obesity as well as a low SES is related to a lower quality of life, which is in line with previous findings revealing that obesity is associated with psychological problems and a poor quality of life (Burkert et al., 2012, 2013a, 2013b; Kinge & Morris, 2010; Stranges et al., 2014), and that people with a low SES show unfavorable psychosocial factors (Burkert, Rásky & Freidl, 2012; Burkert et al., 2012, 2013a, 2013b; Hoebel et al., 2017; Marmot, 2007; Marmot & Wilkinson, 2005; The Marmot Review, 2010) as well as a higher prevalence of mental health problems (Huurre et al., 2007; Reiss, 2013; Van Der Waerden et al., 2014).

The strengths of the study include analyses based on a representative sample of the Austrian population which allows drawing conclusion concerning public health interventions which should be prioritized. Moreover, data was analyzed stratified by sex and corrected for age of the participants, and all variables were measured in a standardized way. Potential limitations of our results are due to the fact that the survey was based on cross-sectional data. Therefore, no statements can be made concerning a causal relationship, but only describe ascertained associations. Hence, further longitudinal studies are required to substantiate our results. Further limitations include the measurement of some variables (e.g., eating behavior or physical activity) as a self-reported and not validated variable. Besides being overweight, the amount of adipose tissue and its location determines the health risk (Kiefer et al., 2006). Unfortunately, only body weight and height were covered by self-reporting at the ATHIS. Therefore, further studies which also measure the waist circumference are needed. Moreover, a potential limitation is the fact that the questions in the ATHIS 2014/2015 are not the same as in 2006/2007. Therefore, comparisons could only be made at a limited level.

## Conclusions

Overall, barely half of the Austrian population has a BMI of normal weight, a fact which has no changed in the past 10 years, besides the circumstance that with rising BMI the health risk also increases. To summarize, our study could show a poor health-related behavior and health as well as less quality of life in women and men with a low SES and those being overweight or obese. Most important, our findings provide evidence regarding a larger impact of social inequality in health and quality of life, especially in women. Up until now, hardly any study has analyzed this relationship in Austria for women and men. The gender differences regarding social inequality gradients have important implications, including the fact that obesity is often transmitted from mothers to their children, especially in those with a low SES. This, in turn includes fewer chances to climb up socially since obesity is also linked to socioeconomic disadvantages (Devaux & Sassi, 2013).

The main implication of our findings is the necessity to consider socioeconomic factors when calculating the health risk. Since our findings revealed a larger social impact in health and quality of life, public health programs which include an interdisciplinary approach are needed to reduce the health risk associated with social inequality in obesity.

## Supplemental Information

10.7717/peerj.6773/supp-1 Supplemental Information 1 ATHIS 2015 SPSS data. Calculations were made with the data in column 481–567. The appropriate data description was tranlated into english.

## Additional Information and Declarations

### Data Availability

The following information was supplied regarding data availability: The raw measurements are available in the Supplemental File. The ATHIS has been collected by the Austrian Statistical Agency and is provided for free use to researchers of scientific institutions. Analyses were calculated by using the data in column 534–569 (with variables which have been calculated based on the raw data).
